# The Protean *Acremonium*. *A. sclerotigenum/egyptiacum*: Revision, Food Contaminant, and Human Disease

**DOI:** 10.3390/microorganisms6030088

**Published:** 2018-08-16

**Authors:** Richard C. Summerbell, Cecile Gueidan, Josep Guarro, Akif Eskalen, Pedro W. Crous, Aditya K. Gupta, Josepa Gené, Jose F. Cano-Lira, Arien van Iperen, Mieke Starink, James A. Scott

**Affiliations:** 1 Sporometrics, 219 Dufferin St. Ste. 20C, Toronto, ON M6K 1Y9 Canada; 2 Dalla Lana School of Public Health, University of Toronto, Toronto, ON M5T 3M7, Canada; james.scott@utoronto.ca; 3 Australian National Herbarium, National Research Collections Australia, CSIRO-NCMI, Canberra, ACT 2601, Australia; Cecile.Gueidan@csiro.au; 4 Unitat de Micologia, Facultat de Medicina i Ciencies de la Salut and IISPV, Universitat Rovira i Virgili, Reus, 43201 Tarragona, Spain; josep.guarro@urv.cat (J.G.); josepa.gene@urv.cat (J.G.); josep.cano@urv.cat (J.F.C.-L.); 5 Department of Plant Pathology, University of California Davis, Davis, CA 95616, USA; aeskalen@ucdavis.edu; 6 Westerdijk Fungal Biodiversity Institute, P.O. Box 85167, 3508 AD Utrecht, The Netherlands; p.crous@westerdijkinstitute.nl (P.W.C.); a.iperen@westerdijkinstitute.nl (A.v.I.); m.starink@westerdijkinstitute.nl (M.S.); 7 Division of Dermatology, Department of Medicine, University of Toronto, Toronto, ON M5G 2C4, Canada; agupta@execulink.com; 8 Mediprobe Research Inc., London, ON N5X 2P1, Canada

**Keywords:** Hypocreales, biodeterioration, Bionectriaceae, *Acremonium*, *Acremonium sclerotigenum*, *Acremonium egyptianum*

## Abstract

*Acremonium* is known to be regularly isolated from food and also to be a cause of human disease. Herein, we resolve some sources of confusion that have strongly hampered the accurate interpretation of these and other isolations. The recently designated type species of the genus *Acremonium*, *A. alternatum*, is known only from a single isolate, but it is the closest known relative of what may be one of the planet’s most successful organisms, *Acremonium sclerotigenum/egyptianum*, shown herein to be best called by its earliest valid name, *A. egyptiacum*. The sequencing of ribosomal internal transcribed spacer (ITS) regions, actin genes, or both for 72 study isolates within this group allowed the full range of morphotypes and ITS barcode types to be elucidated, along with information on temperature tolerance and habitat. The results showed that nomenclatural confusion and frequent misidentifications facilitated by morphotaxonomy, along with misidentified early sequence deposits, have obscured the reality that this species is, in many ways, the definitive match of the historical concept of *Acremonium*: a pale orange or dull greenish-coloured monophialidic hyphomycete, forming cylindrical, ellipsoidal, or obovoid conidia in sticky heads or obovoid conidia in dry chains, and acting ecologically as a soil organism, marine organism, plant pathogen, plant endophyte, probable insect pathogen, human opportunistic pathogen, food contaminant, probable dermatological communicable disease agent, and heat-tolerant spoilage organism. Industrially, it is already in exploratory use as a producer of the antibiotic ascofuranone, active against trypanosomes, cryptosporidia, and microsporidia, and additional applications are in development. The genus-level clarification of the phylogeny of *A. egyptiacum* shows other historic acremonia belong to separate genera, and two are here described, *Parasarocladium* for the *Acremonium radiatum* complex and *Kiflimonium* for the *Acremonium curvulum* complex.

## 1. Introduction

*Acremonium* is one of the genera selected for this Special Issue, as it is well established as a potentially food-borne organism that also plays a role in infectious diseases of humans and animals [1]. The study of overlapping biotypes commonly isolated from food and cases of human disease soon led to a focus on one particular group of *Acremonium* species as significant in these areas. The sequence analysis of DNA loci was needed to clarify the circumscription of the group of interest.

Molecular phylogeny is particularly revelatory in the case of simply structured organisms, which produce few morphological clues to their natural affinities. Phylogenetic revisions [2,3,4] of the species grouped in the morphogenus *Acremonium* by Gams [5] contained at least two major surprises. One surprise was that multiple families and orders of fungi were encompassed in the concept. Indeed, species of radically different phylogenetic affinity could look almost indistinguishable; an example is the convergence of the Hypocrealean *Acremonium sclerotigenum* (particularly mucoid-conidial isolates not forming sclerotia) and its distant relative *Acremonium* (now *Sarocladium*) *strictum*, as well as the far-removed Plectosphaerellaceous *Acremonium cucurbitacearum*, now *Plectosphaerella melonis*. The other surprise was that conidiogenesis was strongly pleomorphic within some species. Different morphs with identical or near-identical sequences at standard biosystematically used loci had sometimes been described as separate species. In the especially dramatic case of *Acremonium sclerotigenum/egyptiacum*, conidiogenesis, though always phialidic, differed markedly among isolates. Some mainly produced cylindrical to long-ellipsoidal conidia in mucoid heads, while others produced broadly ellipsoidal conidia in heads. There were also many isolates producing obclavate to obovoid conidia in dry chains. The different conidial morphs had distinct colours: the mucoid morphs were pale to medium salmonaceous, while the catenate morphs matured as dull greenish grey. This unexpected plasticity helped to inform a revised view of the taxonomic informativeness of fungal conidiogenesis [6].

The long-standing practical consequence of this sort of plasticity was that very few historic identifications of the *Acremonium* species from substrates of practical interest, like food, could be trusted. The exceptions were a few cases where voucher isolates were retained and could later be sequence-identified. The small number of such isolates in collections indicated that the name *Acremonium sclerotigenum* was sometimes isolated from contaminated food [7]. The species concepts had to be redefined, however, in order to be certain which isolates, if any, should truly bear this name, particularly as early sequencing had deposited many reference sequences under names known to be incorrect. The phylogenetic analysis used to clarify species definitions also frequently brought the names of genera into question, and further biosystematic decisions had to be made at that level.

The morphological convergence among *Acremonium* types was so extensive that, prior to 2011, there were at least five major, phylogenetically distinct contenders among sequence types for the role of epitype of *Acremonium alternatum*, the type species. This typification was only substantiated by a scant dried type specimen and illustrations. The choice of epitype would root the future phylogenetic concept of *Acremonium*. Even when the contenders were vetted by exact morphological comparison with Gams’ illustrations of dried type material [8], as well as habitat considerations (leaf litter, near Rostock, Germany), the name could not be narrowed down to a single phylogenetic species. At the time, the most economically, medically, and industrially important species in *Acremonium* were (a) those related to *A. kiliense* and *A. strictum* (two closely related species), (b) the antibiotic producers *A. chrysogenum* and *Emericellopsis salmosynnemata* (which had an *Acremonium* asexual mvorph), and (c) *Acremonium sclerotigenum*. The importance of *A. sclerotigenum* was masked by circumstance: its morphological diversity meant that most isolates had been identified under other names, usually *A. strictum* or *A. potronii* for isolates with mucoid conidia and *A. egyptiacum* or *A. alternatum* for isolates with catenate conidia. Sequence evidence revealed that the importance of *A. strictum* as an opportunistic human pathogen was based on misidentification: all such identifications that could be definitively traced were *A. sclerotigenum* or *A. kiliense* [7]. *A. kiliense* was closely related to the type species of a valid genus, *Sarocladium*, originally distinguished by a minor apomorphy, slightly penicillate conidiophore branching. It was not especially closely related to any candidate for *A. alternatum*, nor were *A. chrysogenum* or *E. salmosynnemata*.

Meanwhile, sequenced isolates revealed that *A. sclerotigenum* was going unrecognised in many important roles and microhabitats that were typical of existing concepts of *Acremonium*. Though not infrequently identified as *A. alternatum* itself when catenate, it had a distinct grey-green tone in chained conidial masses that was not mentioned in the description of the type. It was, however, closely related to an isolate that was an excellent candidate for ‘the real’ *A. alternatum* in every way, CBS 407.66 (CBS = Westerdijk Institute, Utrecht, the Netherlands). For all these reasons (and others), *A. kiliense* and *A. strictum* were recombined into *Sarocladium*, and *A. alternatum* was epitypified with CBS 407.66 with the express aim of including *A. sclerotigenum* in the core group of the revised genus *Acremonium*.

The object of the present paper is to document the intrinsic phylogenetic biodiversity, protean ecology, multifaceted anthropic significance, and complex nomenclature of this archetypical *Acremonium* species. For practical purposes, in reference to the theme of this Special Issue, this clarification is expected to provide an essential underpinning for interpreting *Acremonium* isolations, as well as nucleic acid and secondary metabolite data, from food-related and biomedical sources. Without such a study, given the prevalence of misidentified reference sequences in public databases and the many known reports already marred by use of these inappropriate names [7], authors remain at risk of inadvertently generating data that are misleading or arduous to interpret.

## 2. Materials and Methods

Isolates were obtained from the collections of the Westerdijk Institute (previously called the CBS Fungal Biodiversity Centre; herbarium designation still CBS) and the FMR collection (Facultad de Medicina, Reus, Tarragona, Spain). Many of the latter isolates had originally been received from the University of Texas Health Science Center at San Antonio, TX, USA, and had UTHSC (University of Texas Health Science Center) numbers. Several isolates from the Ontario Ministry of Health (OMH) were tested, and while most were deposited into CBS and listed under those numbers, a few have only OMH numbers.

The isolates were grown for microscopic observation and temperature growth tests on malt extract agar (MEA; 3% malt extract, Oxoid, Basingstoke, Hampshire, UK) and oatmeal agar (OA; [9]). Growth at human body temperature (37 °C) was monitored at 7 and 14 days for selected isolates considered to represent the range of morphological diversity seen in the isolates.

The DNA for the isolates handled at the Westerdijk Institute was extracted with a FastDNA kit (Qbiogene, Heidelberg, Germany) from mycelium grown for 5–14 days in liquid complete medium [10]. One mL of each DNA sample (containing 50–100 ng DNA mL^–1^) was subjected to PCR amplification. The nuclear ribosomal internal transcribed spacer (ITS) region was amplified (ITS1 spacer, 5.8S ribosomal region, ITS2 spacer) using primers ITS1 and ITS4 as described by White et al. [11].

The amplicons were cleaned using the GFX PCR DNA Purification Kit (Amersham Pharmacia, Little Chalfont, UK) as per the kit’s instructions. The cleaned DNA was subjected to cycle sequencing using the same primers used for PCR. The cycle sequencing program was as follows: 25 cycles of 95 °C (20 s), 50 °C (15 s), and 60 °C (60 s). The cycle sequencing PCR products were purified using Sephadex G-50 (Amersham Pharmacia) and sequenced at the Westerdijk Institute using BigDye chemistry and an ABI 3730xl sequencer (Applied Biosystems, Carlsbad, CA, USA).

At FMR, total genomic DNA was extracted from colonies grown on potato dextrose agar (PDA; 4 g of potato infusion, 20 g dextrose, 15 g of agar-agar, 1 L tap water) after 7 days of incubation at 20 ± 1 °C, using the FastDNA kit protocol (per Bio101, Vista, CA, USA), with a FastPrep FP120 instrument (Thermo Savant, Holbrook, NY, USA) following the manufacturer’s protocol. The DNA was quantified using the Nanodrop 2000 (Thermo Scientific, Madrid, Spain). The ITS region and a fragment of the actin gene were amplified with the primer pairs ITS1/ITS4 [11] and ACT1/ACT4R [12], respectively. The amplicons were sequenced in both directions with the same primer pair used for amplification at Macrogen Europe (Macrogen Inc., Amsterdam, The Netherlands). The consensus sequences were obtained using the SeqMan software version 7.0.0 (DNAStar Lasergene, Madison, WI, USA).

At the Australian National Herbarium, the Lasergene SeqMan and EditSeq software modules (DNAstar Lasergene) were used to assemble and edit the sequence files. The sequences were then aligned manually in Mesquite version 3.40 [13]. The two gene regions were analysed separately. The ITS dataset included a total of 72 isolates, and six of these (four species of *Emericellopsis*, one species of *Stilbella*, and the ex-type strain of *Acremonium tubakii*) were used to root the tree. The actin dataset included a total of 49 taxa, with *Acremonium curvulum*, *Acremonium spinosum*, and *Emericellopsis glabra* as outgroup taxa. A GTRCAT model was applied to the two markers, and the two datasets were analysed using maximum likelihood (ML) with the software RAxML VI-HPC v.8.2.9; [14,15]), as implemented on the CIPRES Web Portal (http://www.phylo.org; [16]). Support values were obtained using a fast bootstrap analysis of 1000 pseudoreplicates. The trees were visualised in PAUP* [17] and edited with Illustrator (Adobe Systems, San Jose, CA, USA). The datasets were deposited in TreeBase (accession 22890), sequences in GenBank, and nomenclature in MycoBank.

## 3. Results

DNA sequencing at both the ITS and actin loci showed that isolates with a range of morphologies were grouped into a cohesive sequence group. Within this group, ITS sequences were consistently subdivided into three minimally delimited genetic variants. The sequence differences for these ITS barcode variants are shown in Table 1. A small number of sequences within each barcode type had other minor divergences, not consistent from strain to strain.

**Figure 1:**
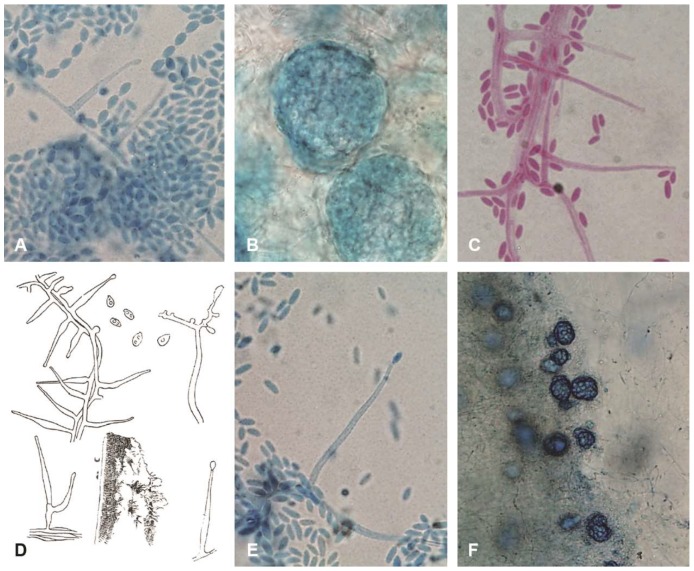
Morphological diversity of *Acremonium sclerotigenum/egyptiacum* plus a comparison with an illustration of the original *Acremonium potronii*. (**A**–**E**) ×1000, (**F**) ×400. Blue structures stained in lactic-acid-cotton-blue stain, reddish in lactofuchsin: (**A**) CBS 545.89, catenate-conidial isolate originally identified as *A. alternatum*; (**B**) CBS 545.89 sclerotia; (**C**) CBS 251.95 mucoid-conidial isolate with ellipsoidal conidia, originally identified as *A. potronii*; (**D**) original Vuillemin drawing of *A. potronii*; (**E**) CBS 113276, typical mucoid-conidial *A. sclerotigenum* isolate with cylindrical conidia; and (**F**) heavily sclerotial Iranian isolate Zare 60-#3.

A select group of isolates at CBS and at FMR were studied to analyse morphological characters. A smaller group of isolates held at CBS were tested for growth at 37 °C. Figure 1A–C,E,F show the range of morphologies seen: catenate conidia of CBS 545.89 (Figure 1A) consistent with Gams’ concept of *A. alternatum*, ellipsoidal, mucoid conidia of CBS 251.95 (Figure 1C) consistent with Gams’ concept of *A. potronii*, and cylindrical, mucoid conidia of CBS 113276 (Figure 1E) consistent with the original description of *A. sclerotigenum*. Sclerotia are shown both for the catenate-conidial 545.89 (Figure 1B) and at lower magnification for the mucoid-conidial Zare 60-#3. The original 1910 line-drawing of *Acremonium potronii* (Figure 1D), reproduced here for comparison, is described in the Discussion.

In Table 2, the characteristics of the isolates studied in detail are listed with the identification and isolation details of all the other isolates in this study for which ITS sequences were obtained. Table 3 lists isolation details for isolates included in actin sequencing but not elsewhere in the study. It also lists details for outgroup isolates used in analysis in the ITS and actin studies.

Table 2 shows that among the three common ITS barcode types, only the first one, 33C/106C/388C, was predominantly seen in isolates obtained outside clinical (including medical and veterinary) environments. Of 32 such isolates, 23 were from environmental sources, eight from clinical sources, and one of unrecorded origin. In contrast, for ITS barcode 2 (33T/106C/388C), six of 11 isolates were from clinical sources. For barcode type 3, (33T/106T/388A), only two of 19 isolates were known to be from environmental sources, while just one was from an unrecorded source, leaving the remaining 16 clinical. Each barcode type contained an isolate involved in a published medical or veterinary case: lethal infection of an ostrich egg in barcode 1 [18], dialysis-related peritonitis in barcode 2 [19], and systemic infection of an immunocompromised patient in barcode 3 [20]. Most isolates obtained from humans were from infected toenails. Many were from repetition-confirmed infections as described by Gupta et al. [21]; the 32 “*Acremonium* sp.” isolates listed in that publication as confirmed causes of onychomycosis were all later identified as *A. sclerotigenum*.

The isolates from plant sources were diverse and mostly not ecologically interpretable, but barcode types 1 and 2, particularly the former, were regularly isolated as endophytes of grapevines, including numerous isolates from California vine arms obtained by one of us (A.E.).

The isolates derived from food-related sources were all of barcode type 1 and were obtained from fishmeal, cucumber, muskmelon, and a grapefruit juice can. Whether the many isolates from grapevines have any connection to the quality of grapes as food was not investigated.

Studies of growth at 37 °C revealed that many of the isolates in barcode types 1 and 2 could survive 14 days at body temperature but could not grow. In each barcode type, at least one isolate was seen that could grow minimally off the inoculum plug into the surrounding MEA during 14 days at 37 °C. All three test isolates of barcode group 3 grew at least minimally at 37 °C, and isolate 287.70O from Egyptian soil grew 9 mm in diameter in 14 days.

The majority of the catenate-conidial isolates conforming to the prototype *Acremonium egyptiacum* morphology belonged to barcode type 1, but one such isolate, CBS 545.89, transpired to belong to barcode type 2. Perdomo et al. [4] reported that an isolate consistent with barcode type 3, UTHSC 05-1172, formed conidia in chains, but that isolate was subsequently lost, and no similar isolate has been detected so far.

**Figure 2:**
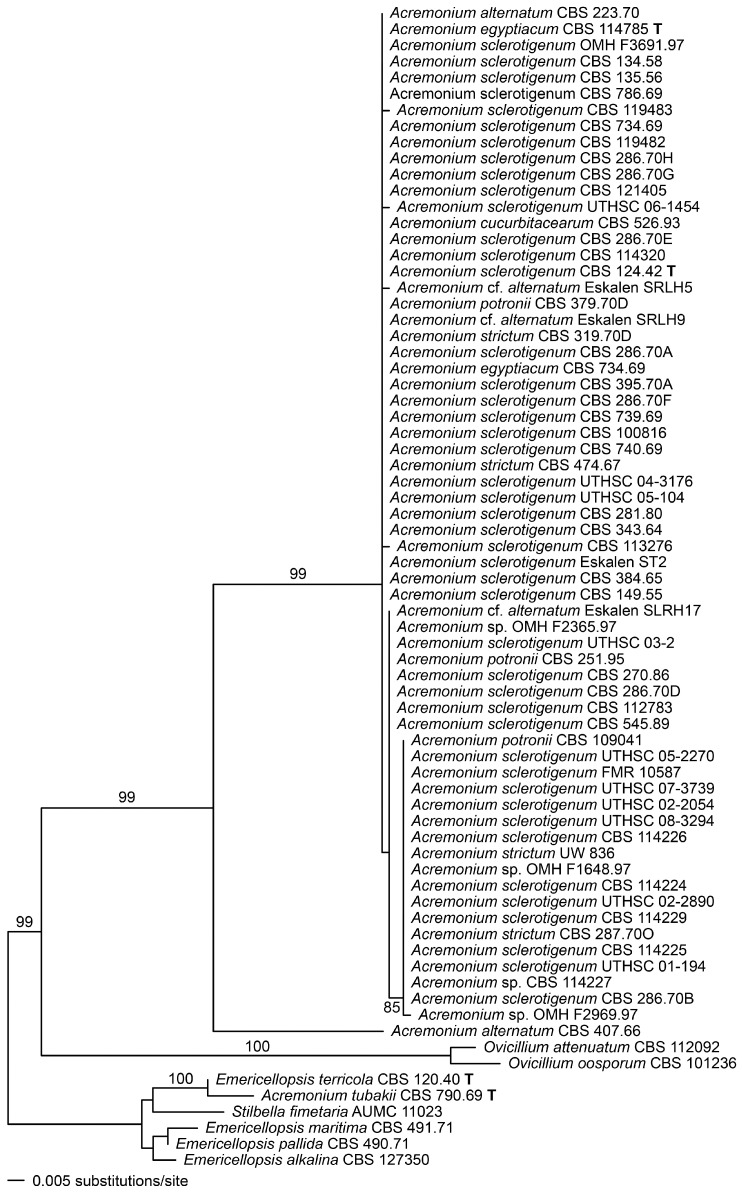
Phylogenetic disposition of the *Acremonium sclerotigenum/egyptiacum* complex as shown by complete ribosomal internal transcribed spacer (ITS) sequences analysed using maximum likelihood (ML) with RAxML VI-HPC. Support values were obtained using a fast bootstrap analysis of 1000 pseudoreplicates. Members of the *Emericellopsis-Stilbella* complex are used as an outgroup.

**Figure 3:**
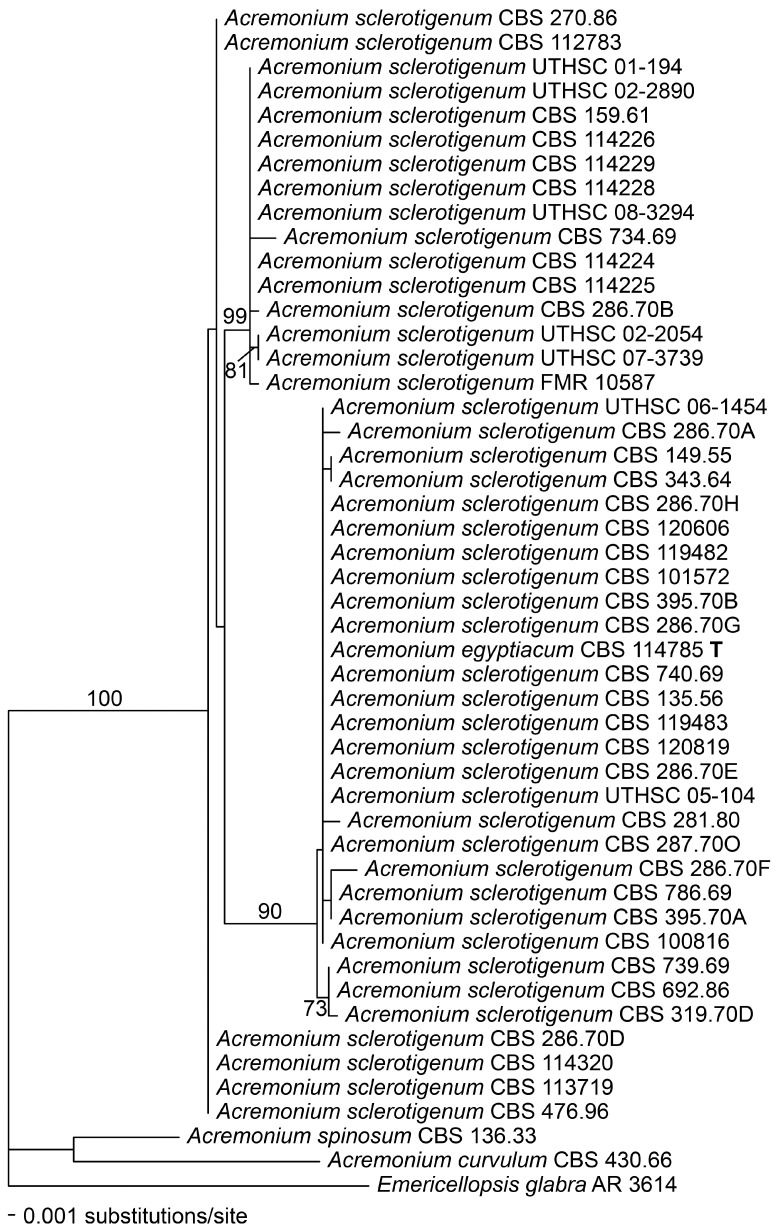
Phylogenetic disposition of the *Acremonium sclerotigenum/egyptiacum* complex as shown by a fragment of the actin gene amplified using the primers ACT1 and ACT4R, and analysed using maximum likelihood (ML) with RAxML VI-HPC. Support values were obtained using a fast bootstrap analysis of 1000 pseudoreplicates. *Acremonium spinosum*, *A. curvulum*, and *Emericellopsis glabra* appear as outgroups.

Alignments of the ITS and actin regions showed that ITS barcode types were minimally resolved, although in the ITS dendrogram itself (Figure 2), the barcode type 2 isolates tended to cluster together, and the barcode type 3 isolates made up a distinct branch. In the actin dendrogram (Figure 3), the ITS barcode type 3 isolates again mostly clustered together on a unique branch, but there were two anomalies. The branch containing the type 3 isolates also consistently contained, on a short side-branch, CBS 734.69, an ITS type 1 strain with catenate conidiation isolated from tomato roots in Turkey. Also, CBS 287.70O, an ITS type 3 isolate from Egyptian soil, did not cluster with other type 3 isolates in actin analysis but instead clustered among the type 1 and 2 isolates.

Figure 2 and Figure 3 also show that the genus *Acremonium* sensu stricto (s. str.), as represented by its apparently rare type species, *A. alternatum*, and the common *A. sclerotigenum*/*egyptiacum* group, is well demarcated from the most closely situated genus in ITS analysis, the recently described *Ovicillium*, composed of isolates conforming to the outdated *Verticillium* sect. *Albo-Erecta*. Also distinctly delimited is the cluster containing *Stilbella* s. str. and *Emericellopsis* (along with the closely associated *Stanjemonium*, not shown). Other related genera, *Geosmithia* and *Clonostachys*, are too distant to align reliably in the ITS analyses used here, and few compatible actin sequences exist for related fungi.

## 4. Discussion

### 4.1. Taxonomy and Nomenclature of the Acremonium sclerotigenum/egyptiacum Cluster

As Perdomo et al. [4] noted, there is a need to settle the matter of the correct naming of the unexpectedly phenotypically diverse group of isolates encompassed in the *A. sclerotigenum/egyptiacum* cluster. The balance of the Discussion will be most comprehensible if this matter is settled before other matters are addressed.

We conclude that the ITS and actin sequences used do not support the naming of additional species or infraspecific taxa in this group. Apparent contradictions between ITS and actin placements of a few strains suggest that a sexual or hybridisation process may be shuffling markers within the isolates studied and their conspecifics. Although this re-assortment might well indicate linkage disequilibrium within a sexual species, the differences are small enough that chance alone may also be a factor.

Despite their morphological differences, the isolates ex-type of the original species names *Cephalosporium sclerotigenum* Moreau and R. Moreau ex Valenta 1948 (validation of an invalid name from 1941) and *Oospora egyptiaca* J.F.H. Beyma 1933 are very closely related, both belonging to ITS barcode type 1. The earlier valid name, in its modern generic combination, is thus *Acremonium egyptiacum* (J.F.H. Beyma) W. Gams. Both names are underutilised in the literature, because isolates are so frequently misidentified as *Sarocladium* (*Acremonium*) *strictum* or *A. alternatum*, and there is no eminent economic or cultural momentum that would argue for the junior name being formally conserved against the senior.

The main outstanding question, then, is whether another of the names frequently found in use for this group, *Acremonium potronii* Vuill. 1910, might be the true earliest name. Although the isolate ex-type, illustrated by Vuillemin [22] and later in more detail by Pollacci and Nannizzi [23], has not survived to be analysed genetically, the illustrations show a superficially plausible, *Acremonium sclerotigenum*-like form with ellipsoidal to obovate conidia in mucoid heads (Figure 1D). The isolation is from a medical case possibly involving systemic dissemination (see detailed synopsis by Summerbell [24]). Very few *Acremonium*-like species are confirmed as causing such cases or growing at 37 °C, usually a prerequisite for human infection, but members of the *A. sclerotigenum* group have been shown to do both. Close examination of Vuillemin’s illustrations and descriptions, however, reveal that nearly all of the phialides he drew for *A. potronii* were adelophialides—that is, they lacked a basal septum. Some of them were foreshortened to minute phialidic necks protruding laterally along hyphae, while others were fully developed aculeate structures, frequently somewhat swollen between the basal region and the mid-region. The drawings were of impeccable accuracy; Vuillemin, in the same publication [22], coined and defined the term ‘phialide’ for the first time, giving diverse well-scrutinised examples, and he specifically excluded the structures seen in *A. potronii* from that term because they were not delimited from the subtending hypha [25]. Gams [5] accepted *A. potronii* as a name for *Acremonium* types whose phialides all had basal septa, and he identified several *A. sclerotigenum*-group isolates with this name (e.g., CBS 251.95), although none of the three *A. potronii* isolates illustrated in his 1971 monograph is a member of this group. Based on current research findings, it appears likely that *A. potronii* was a *Phialemonium*-, *Phialemoniopsis*-, or *Coniochaeta*-like fungus [26,27]. Its exact identity is unclear, and we consider it a *nomen dubium*.

The correct name for the species studied by us, then, is *Acremonium egyptiacum* (J.F.H. Beyma) W. Gams.

### 4.2. Ecology and Medical and Economic Significance of A. egyptiacum

As detailed by Summerbell and Scott [7], most published medical cases historically attributed to “*Acremonium strictum*” turned out to be caused by *A. egyptiacum* in cases where sequence identification or re-identification became available. Members of ITS barcode group 3 appear to be the most important, causing, to begin with, two well-documented cases of disseminated infection in immunocompromised patients, one attributed by Novicki et al. [20] to ‘*Acremonium strictum* genogroup 2’, and a more recent case attributed by Guitard et al. [28] to ‘*Acremonium sclerotigenum/egyptiacum*’. Numerous onychomycosis cases studied by Gupta et al. [21] also can now be attributed to this subclade [6], and there are many isolates obtained from infected toenails in other regions (Table 2 and Table 3), though not all are repetition-confirmed as true etiologic agents [29]. This subclade is isolated so regularly from toenail onychomycosis, mostly in the elderly, that it may be hypothesised to have developed an ongoing niche as a communicable disease of the nails. If it were environmentally common, as are other nail infecting non-dermatophyte fungi like *Scopulariopsis brevicaulis*, it could be interpreted as a regular invader of nails from other environmental substrates; but, its relatively rare environmental isolation suggests that it may instead be transmitted from person to person via transient environmental inoculum as are dermatophytes. This would make its ecology comparable to that of *Neoscytalidium dimidiatum*, which has wild-type subtypes that are seen both in woody plant infections and in human skin and nail infections, as well as divergent subtypes, featuring slow growth or loss of melanisation, which are only known from dermatological infections [30].

The presence of *A. egyptiacum* in the nails may become a systemic hazard if the patient becomes immunocompromised: the case of blood-borne disseminated infection studied by Guitard et al. [28] appeared to arise from a toenail with a prior *A. egyptiacum* infection.

A case of prosthetic mitral heart valve infestation documented by Guarro et al. [31] can now be attributed to *A. egyptiacum* ITS barcode type 3, based on its sequence, in GenBank as AM990178.

The relative virulence of two ITS barcode type 3 isolates, UTHSC 01-194 and 05-2270, both originally from blood, was studied in an immunocompromised mouse model by Fernández-Silva et al. [32]. The isolates proved only to infect the immunosuppressed mice, but not immunocompetent controls, and killed 70% of the susceptible animals within 16 days even when low inoculum levels were used.

Barcode type 2 can be credited with a case of kidney dialysis-related peritonitis in a Greek patient [19]. The isolate was kindly forwarded to CBS and is included in this study as CBS 112783.

Barcode type 1 isolates have been isolated from toenails, and there is an isolate, UTHSC 04-3176, listed as derived from cerebrospinal fluid. This is a normally sterile bodily material, but even though microorganisms are normally absent in it, medical significance cannot automatically be attributed to common environmental fungi isolated from it. Process contamination, such as microscopic-scale exposure of syringe needle interiors to contaminant fungal spores from room air or alcohol-swabbed skin, cannot be entirely excluded in the making of clinical isolations from normally sterile bodily materials. Confirmation of pathogenicity requires not just isolation alone, but also supportive case information, such as pathology findings or repeated isolations. Thus, UTHSC 04-3176 is of uncertain medical significance. The one well-confirmed case of clinical significance caused by an isolate of this barcode type (specifically, CBS 100816) is an infection of a farm-produced ostrich egg investigated in Italy because it failed to hatch [18].

*A. egyptiacum*, as seen in Table 2 and Table 3, occurs regularly in many areas as an endophyte of grapevines; one of us (R.C.S.) has identified many isolates from this habitat sent in to the CBS identification service but not retained in the collection. The species was also found, as *A. sclerotigenum*, to be a year-round endophyte of *Quercus ilex* in north-central Spain [33]. Barcode type 1 isolates, listed as *Acremonium* sp., were obtained in Korea as an endophyte of the medicinal Chinese boxwood, *Lycium chinense* [34]. *A. egyptiacum* was a moderately prevalent isolate from diseased or dead larvae of the rose stem girdler beetle *Agrilus aurichalcenus* in the Kerman Province of south-eastern Iran [35]. Here, the identifications were not stated as having been confirmed by sequencing, but a photograph shows the recognisable pale greyish green of catenulate isolates of *A. egyptiacum*, and the authors were aware of our as yet unpublished findings about this name. Most isolations from plants are of unknown significance: for example, scattered isolations from the phylloplane of barley (CBS 114320; [36]) may reflect growth in situ or sedimentation from air spora.

A phytopathogenic role for *A. egyptiacum* (as *A. sclerotigenum*) was documented by Li et al. [37], who fulfilled Koch’s postulates after discovering the fungus caused a spot disease on bagged apples. The sequences obtained were likely of barcode type 1, because CBS 384.65 (GenBank HQ232129) was cited as the reference used in identification. Both a morph with mucoid heads and a morph with long conidial chains were involved; they had identical sequences at three loci. This finding may connect with the isolation of CBS 343.64 from lenticel of an apple tree.

Koch’s postulates were again fulfilled for *A. egyptiacum* as a causal agent of rose dieback in Iran’s Fars province [38]. Both isolates sequenced in the study (GenBank KU532330, KU532331) conformed to ITS barcode type 1 and had conidia in mucoid heads. Evidence of significant aerial transmission of inoculum was obtained by spore trapping, possibly indicating that some chains were also formed in situ.

Sequences obtained via denaturing gradient gel electrophoresis (DGGE) from the root zone of the Scrophulariaceous medicinal plant *Rehmannia glutinosa* (‘sheng di huang’ approximately transcribes the common-name 生地黄) in Henan Province, China, were compatible with ITS barcode type 1, with a GenBank ITS sequence of *A. sclerotigenum* ex-type CBS 124.42 (HQ232209) cited as a reference comparison [39]. The authors interpreted these sequences as indications of a potential pathogenic contribution from *A. egyptiacum* (as *A. sclerotigenum*) in a toxic-soil replant problem with the plant.

The correction via sequencing of a long-standing misidentification of an *A. egyptiacum* barcode type 1 isolate (GenBank LC063776) as *Ascochyta viciae* allowed this species (as *A. sclerotigenum*) to be identified as the producer of the meroterpenoid compound ascofuranone, a cyanide-insensitive alternative-oxidase inhibitor with proven efficacy against rodent infections of African trypanosomiasis and cryptosporidiosis [40], as well as laboratory-demonstrated potential against microsporidia [41]. *A. egyptiacum* also produces the related compounds ascochlorin and ascofuranol [42]. Other industrial explorations have shown that the species can affect some potentially useful transformations of pharmaceutically produced steroids [43]. A recent study has stated that an *A. egyptiacum* (identified as *A. sclerotigenum*) isolate obtained from *Flabellia petiolata* marine alga produces amyloidogenic hydrophobin proteins that may be of interest in the manufacture of ‘green’ bio-coatings [44]. However, a search in GenBank for sequences from this study yielded only accession KR014351 for strain MUT-4872; this sequence matches a known undescribed species related to *Acremonium hennebertii*. It may have BLASTed most closely to *A. egyptiacum* sequences.

Correctly identified marine isolates with ITS sequences 99% similar to those of CBS 114785, the ex-type of *A. egyptiacum*, were cited from seawater near the Northern Antarctic Peninsula by Gonçalves et al. [45]. Examination of the sequences showed they were of barcode type 3. “*Acremonium alternatum*” isolates listed as having 99% similarity to GenBank record U57674.1, the ITS sequence of CBS 223.70, were obtained from marine sediments associated with coal deposits [46]. The exact sequences obtained were not deposited. *A. egyptiacum* barcode type 1 was found repeatedly in petroleum-contaminated soils of the Khuzestan Province, southern Iran [47]. Note that GenBank accession KY039283, the first of four sequences listed by these authors as *A. sclerotigenum*, is actually diagnostic of an *Emericellopsis* species, with a characteristic ACAAAACTTT motif at the beginning of the 5.8S rDNA.

Certain isolations, such as that of CBS 149.55 from a rust fungus lesion, raise the possibility that *A. egyptiacum* may be a facultative mycoparasite. There is little evidence for this as yet, but because closely related species, especially *Ovicillium oosporum* Zare and W. Gams, are well-established fungicolous fungi, evidence of this role in *A. egyptiacum* should be watched for. Plant endophytism, for example, may in some cases include or be based upon parasitism of other fungal endophytes.

It is likely that *A. egyptiacum* will be found to be one of the major *Acremonium*-like organisms associated with food spoilage, if not the most important. The species’ association with multiple plant and animal sources, and its ability to grow at relatively high temperatures, in combination with its general morphological compatibility with *Acremonium* sp. contaminants described at the genus level in food-related textbooks, makes this seem likely. There may be medical implications, because the intestine can be a portal of entry for infection in severely immunocompromised patients. A disseminated infection attributed to an untraceable “*Acremonium strictum*” isolate by Schell and Perfect [48] appeared to start in the gut, thus probably originating from food. This isolate was probably either *A. egyptiacum* or *A. kiliense* but was most likely the former, because the latter could generally be distinguished easily in pre-molecular identification by the formation of chlamydospores, and also was well known to produce distinctive melanoid pigment on Sabouraud agar. *A. egyptiacum* has elevated heat tolerance: it is able to survive 90 °C heat treatment for 50 min [49]. Its production of sclerotia probably significantly increases its ability to survive under adverse conditions [38]. This sort of heat tolerance may facilitate the contamination of heat-disinfected foods.

Recently, one of us (R.C.S.) curated the alignment of 170 *A. egyptiacum* sequences assembled in the Plutof Biodiversity Platform (https://plutof.ut.ee/) [50]. Many of these sequences were deposited under incorrect names or as unknown sequences obtained in molecular community studies. Study of the origins of these sequences, and tracking of any published material involved, could shed considerable further light on the ecology of this fungal group. A preliminary survey of the first few items listed yields variously identified barcode type 1 isolates in a wide range of substrata. There are colonisers of *Peniophora* basidiomycetes in Texan attine ant nests (GenBank HQ607928; sequence misidentified as *Peniophora* sp.), as well as endophytes of *Bletilla ochracea* (Chinese butterfly orchid) (HM751796) and *Atractylodes lancea* (Asteraceae) (KC172079) in China. Other isolates are from *Taxus globosa* bark in Mexico (JF773645), and from human nails in China (KT878342, KT878343). Barcode 3 isolates appear as colonisers of human nail (KC254088, MITS56|KP132613) and blood (KC254089|MITS52) in Greece. Others appear as endophytes of *Gossypium* in China (KF999020) and as colonisers of marine macroalgae (HQ914912) and deep-sea materials (KM274118). Already, combined with the results of Gonçalves et al. [45], mentioned above, a possible trend for barcode type 3 to be isolated from marine materials can be perceived.

### 4.3. Broader Taxonomic Situation of the Genus Acremonium

The compilation of information on the biodiversity within *A. egyptiacum* shows that this organism’s range of ecological, clinical, and economic roles epitomises most of the generalities assigned to the genus *Acremonium* in general textbooks, such as the *Introduction to Food- and Airborne Fungi* [51]. Thus, prior concepts of *Acremonium* as a genus are preserved in this phylogenetic nomenclature even though the great majority of species historically given this genus name will be excised. There may be some additional species related to *A. sordidulum* and *A. brachypenium* that will remain within *Acremonium*, but the close phylogenetic proximity of a very different genus, *Ovicillium,* effectively barricades extension of the name beyond the point where *Acremonium* and *Ovicillium* diverge. The next proximal major clade, the *Emericellopsis-Stanjemonium-Stilbella* one, now incorporates a venerable (1900) and famous genus name, *Stilbella*, and potentially unites this generic name with several heretofore excluded organisms forming synnemata, such as *Emericellopsis salmosynnemata* and *E. synnematicola*.

Most of the groups currently containing *Acremonium* names are in clades that need revision to clarify their unity and extension. There are, however, two highly distinct and isolated genus-level clades, as shown by Summerbell et al. [3], that could be rescued immediately from anachronistic nomenclature. The highly distinct nature of these groups extends to the possession of variant forms of the usual AAACTTT motif that begins the 5.8 ribosomal DNA segment throughout the filamentous Ascomycota in fungi ranging from *Acremonium egyptiacum* to *Fusarium*, *Chaetomium*, *Aspergillus*, and *Ochroconis*. The *Acremonium radiatum* clade has CAACTTT, while the *Acremonium curvulum* clade has AAACCTT. These groups are sufficiently distinct that their ITS sequences cannot be meaningfully aligned with those of other groups. Their current taxonomic situation has been determined via the 18S ribosomal and 28S LSU sequences published previously [3], after updating showed no recently deposited sequences have altered their positions of isolation. Both groups require revision to deal with undescribed species in their midst, but we believe that a good beginning would be to focus interest on these orphaned *Acremonium* clades by giving them working generic names.

A. *Parasarocladium* Summerbell, Scott, Guarro, and Crous, *gen. nov*. (MYCOBANK 826815).

Etymology: name derived from ancient Greek παρά (beside, juxtaposed to) and the genus name *Sarocladium*.

Type species: *Parasarocladium radiatum* (Sukap. and Thirum.) Summerbell, Scott, Guarro, and Crous.

Ascomatal morph not observed. Mycelium consisting of hyaline, septate, branched hyphae. Colonies smooth to thinly floccose, with salmonaceous, orange, yellow, or greenish-black reverse colouration. Conidiophores arising laterally from somatic hyphae, erect, cylindrical to subcylindrical, unbranched or less commonly branched, aseptate or septate, smooth, hyaline. Conidiogenous cells phialidic, arising laterally from hyphae or in terminal pairs, or verticils of three, or small monopodially branched tufts of up to four from conidiophores, monophialidic, hyaline, smooth, mostly aseptate but up to two-septate, elongate-ampulliform to subcylindrical, with inconspicuous collarettes. Conidia aseptate, smooth-walled or with chromophilic roughening, ellipsoidal, ovate, bacilliform or fusiform, sometimes slightly curved, forming slimy heads on the phialides. Chlamydospores absent but hyphal swellings may be present. Characteristic CAACTTT motif at 5′ end of 5.8S rDNA.

1. *Parasarocladium radiatum* (Sukap. and Thirum.) Summerbell, Scott, Guarro, and Crous, comb. et stat. nov., MYCOBANK 826816.

Basionym: *Cephalosporium acremonium* Corda var. *radiatum* Sukapure and Thirum. in *Sydowia* 19: 172 (1966).

Acremonium radiatum (Sukap. and Thirum.) W. Gams, Cephalosporium-artige Schimmelpilze: 125 (1971).

Type: CBS 142.62, ITS accession MH424699.

2. *Parasarocladium breve* (W. Gams) Summerbell, Scott, Guarro, and Crous, comb. et stat. nov., MYCOBANK 826817.

Basionym: *Cephalosporium roseum* var. *breve* Sukap. and Thirum. in Sydowia 19: 174, 1966.

≡ Acremonium breve W. Gams, Cephalosporium-artige Schimmelpilze: 60 (1971).

Type: CBS 150.62, ITS accession MH424706.

3. *Parasarocladium gamsii* (Tichelaar) Summerbell, Scott, Guarro, and Crous, comb. nov. MYCOBANK MB 826818.

Basionym: Acremonium gamsii Tichelaar, Acta Botanica Neerlandica 21: 197 (1972).

Type: CBS 726.71, ITS accession MH424707.

Known members of this genus have been almost exclusively isolated from soil or from possible conidial dispersal media such as air. B. *Kiflimonium* Summerbell, Scott, Guarro, and Crous, gen. nov., MYCOBANK 826819.

Etymology: Named for the resemblance of the curved shapes of the conidia to crescent-shaped pastries known in many areas by the originally Hungarian name kifli (singular noun form), which is adopted as English in some extended dictionaries such as Wiktionary.org; also known by the cognate Serbian, Bosnian, Macedonian, and Bulgarian name *kifla* and the Austrian *kipfel*. (The basic Latin and Greek roots for ‘curved,’ ‘crescent-shaped,’and so forth, are overused in other fungal names, and members of this group of simply structured organisms share no distinctive visible feature other than their curved conidia). -Monium, meaning ‘little singularity,’ derives from *Acremonium*.

Type species: *Kiflimonium curvulum* (W. Gams) Summerbell, Scott, Guarro, and Crous.

Ascomatal morph not observed. Mycelium consisting of hyaline, septate, branched hyphae. Colonies pale to yellow-orange, sometimes producing brown reverse pigments. Conidiophores mostly short side branches arising laterally from somatic hyphae, subcylindrical, unbranched, or branched and bearing mostly 1–4 conidiogenous cells, smooth, hyaline. Conidiogenous cells phialidic, arising laterally and singly from hyphae or on conidiophores in subverticillate clusters of mostly 2–4, mostly monophialidic, hyaline, smooth, generally aseptate, elongate-subcylindrical to aciculate, gently tapered, with inconspicuous collarettes; short, adelophialidic side-branches may be present. Conidia 0–1 septate, smooth-walled, lunate to falcate with rounded or acute ends, or in some cases ellipsoidal; in all cases, forming slimy heads on the phialides. Chlamydospores absent. Characteristic AAACCTT motif at 5′ end of 5.8S rDNA.

1. *Kiflimonium curvulum* (W. Gams) Summerbell, Scott, Guarro, and Crous, comb. nov. MYCOBANK MB 826820

Basionym: Acremonium curvulum W. Gams, Cephalosporium-artige Schimmelpilze: 57, 1971.

No *Kiflimonium* isolate studied so far has been found to grow at 37 °C. Some of the features in the generic description are only known from as yet undescribed organisms stored at CBS.

Members of this genus are mostly isolated from soil; CBS 214.70, distinct by possessing many septate conidia [5], was isolated from rust-infected *Lolium* ryegrass. Isolates accepted as *K. curvulum* ss. str. include (with ITS sequence accessions as substantiation) CBS 430.66 (ex-type; MH424698), CBS 229.75 (MH424700), CBS 101442 (MH424701), CBS 333.92 (MH424702), CBS 898.85 (MH424703), CBS 384.70A (MH424704), and CBS 761.69 (MH424705).

There are at least 10 other major clades of *Acremonium* species that will require new generic names or will need to be recombined into genera originally erected for ascomatal species or for ‘charismatic apomorphs’ (i.e., groups drawing attention with distinctive, evolutionarily derived conidiophore types [3]—cf. *Ovicillium, Stilbella, Trichothecium, Sarocladium*). Species that will be affected by name changes include some very recently described taxa, such as *Acremonium asperulatum* and *A. variecolor* [52], which may belong to *Bulbithecium* or an as yet unnamed neighbour-genus, and *A. pilosum*, *A. parvum*, and *A. citrinum*, which are situated in the phylogenetically isolated *Acremonium fusidioides* clade [53]. We would encourage authors not to hesitate to give simply structured, phylogenetically distinct organisms generic names; hesitation to await discovery of distinctive phenetic features would be an error in scientific strategy. The ecological and biochemical significance of the phylogenetically unified, morphologically flexible concept of *Acremonium egyptiacum* illustrates an important reality: the impact of *Acremonium*-like taxa can only be obscured if biosystematists place too great a focus on morphological delimitation.

## Figures and Tables

**Table 1:** Major variants in internal transcribed spacer (ITS) sequences among isolates identified as conspecific with the type of *Acremonium sclerotigenum.*

ITS Group	Position 33 after Common Startpoint ‘ATCATTA’	Position 106	Position 388
Ex-type strains ^1^ group (Group 1)	C	C	C
Group 2	T	C	C
Group 3	T	T	A

^1^ Contains sequences from ex-type isolates of *Acremonium sclerotigenum* and *A. egyptiacum.*

**Table 2:** Isolates in the *Acremonium sclerotigenum*/*egyptiacum* complex typed by ITS barcode type.

Isolate # (CBS Unless Otherwise Indicated) and GenBank Numbers: ITS, (Actin)	Original Identification (as Received by Us; Nomenclature Not Updated)	ITS Geno-Group	Morphology	Habitat	Origin	Remarks
124.42 MH424620	*Acremonium sclerotigenum* (T)	1	mucoid	sand dune under *Ammophila*	France, region Pays de la Loire	slow 37 °C growth
114785 MH424616, (MH427920)	*Acremonium egyptiacum* (T) as *Oospora egyptiaca*	1	catenate	soil	Cairo, Egypt	
474.67 MH424661	*Acremonium strictum*	1	mucoid	banana leaf in greenhouse	Netherlands	slow growth 37 °C
734.69 MH424615, (MH427921)	*Acremonium egyptiacum*	1	catenate	tomato root	Izmir Prov., Turkey	survives 37 °C, does not grow. Forms sclerotia
319.70D MH424660, (MH427955)	*Acremonium strictum*	1	mucoid	chamois dung	Tyrol, Austria	survives 37 °C, does not grow
526.93 MH424614	*Acremonium cucurbitacearum*	1	mucoid	Muskmelon (*Cucumis melo*)	Spain	slow growth 37 °C
223.70 U57674	*Acremonium alternatum*	1	catenate	wall	France	survives 37 °C, does not grow
100816 MH424640, (MH427956)	*Acremonium sclerotigenum*	1	mucoid	ostrich egg	Piedmont, Italy	Illustrated by Isaia et al. [18]
384.65 MH424635	*Acremonium sclerotigenum*	1	mucoid	human toenail	Québec, Canada	
113276 MH424641	*Acremonium sclerotigenum*	1	mucoid	human	Germany	survives 37 °C, does not grow
OMH F3691.97 MH424648	*Acremonium sclerotigenum*	1	mucoid	human toenail	Ontario, Canada	
Eskalen ST2 MH424646	*Acremonium alternatum*	1	mucoid	grapevine	Madera Cty., California, USA	
Eskalen SRLH9 MH424612	*Acremonium alternatum*	1	mucoid	grapevine	Madera Cty., California, USA	
Eskalen SRLH5 MH424611	*Acremonium alternatum*	1	mucoid	grapevine	Madera Cty., California, USA	
Eskalen SRLH14 MH729059	*Acremonium alternatum*	1	mucoid	grapevine	Madera Cty., California, USA	
UTHSC 04-3176 MH424653	*Acremonium sclerotigenum*	1	mucoid	human cerebro-spinal fluid	Minnesota, USA	
UTHSC 06-1454 MH424656, (MH427916)	*Acremonium sclerotigenum*	1	mucoid	human toenail	Florida, USA	
UTHSC 05-104 MH424654, (MH427917)	*Acremonium egyptiacum*	1	catenate	human, unknown site	California, USA	
786.69 MH424639, (MH427931)	*Acremonium sclerotigenum*	1	mucoid	xylem of *Gossypium*	Iran	
134.58 MH424621	*Acremonium sclerotigenum*	1	mucoid	*Cucumis sativus*	Netherlands	
135.56 MH424622, (MH427934)	*Acremonium sclerotigenum*	1	mucoid	*Anthurium* leaf	Netherlands	
281.80 MH424625, (MH427940)	*Acremonium sclerotigenum*	1	mucoid	human toenail	Netherlands	
119482 MH424643, (MH427923)	*Acremonium sclerotigenum*	1	mucoid	grapevine	Madera Cty., California, USA	
119483 MH424644, (MH427922)	*Acremonium sclerotigenum*	1	mucoid	grapevine	Yolo Cty., California, USA	
286.70H MH424633, (MH427924)	*Acremonium sclerotigenum*	1	mucoid	haversack	Florida, USA	
286.70G MH424632, (MH427925)	*Acremonium sclerotigenum*	1	mucoid	grapefruit juice can	Florida, USA	
121405 MH424645	*Acremonium egyptiacum*	1	catenate	grapevine	Syria	
149.55 MH424623, (MH427933)	*Acremonium sclerotigenum*	1	mucoid	*Puccinia rubigo-vera* uredinium	Germany	
343.64 MH424634, (MH427936)	*Acremonium sclerotigenum*	1	mucoid	lenticel in apple	Finistère Dept., France	
286.70E MH424630, (MH427927)	*Acremonium sclerotigenum*	1	mucoid	ND ^1^	Louisiana, USA	
740.69 MH424638, (MH427932)	*Acremonium sclerotigenum*	1	mucoid	fishmeal	France, Nord Dept.	
286.70A MH424626, (MH427930)	*Acremonium sclerotigenum*	1	mucoid	*Pteridium aquilinum* petiole	Ischia, Italy	
270.86 MH424624, (MH427911)	*Acremonium sclerotigenum*	2	mucoid	human toenail	Nancy, France	
112783 MH424670, (MH427947)	*Acremonium strictum*	2	mucoid	peritoneal fluid	Thessaloniki, Greece	
251.95 MH424617	*Acremonium potronii*	2	mucoid	human nail	Limburg, Netherlands	slow growth 37 °C
545.89 MH424636	*Acremonium alternatum*	2	catenate	human, blood	South Holland, Netherlands	survives 37 °C, does not grow
379.70D MH424618	*Acremonium potronii*	2	mucoid	human foot skin	North Holland, Netherlands	
Eskalen SRLH17 MH424613	*Acremonium alternatum*	2	mucoid	grapevine	Madera Cty., California, USA	
UTHSC 03-2 MH424652	*Acremonium sclerotigenum*	2	mucoid	human sinus	California, USA	
286.70F MH424631, (MH427926)	*Acremonium sclerotigenum*	2	mucoid	ND	Florida, USA	
114320 MH424642, (MH427937)	*Acremonium sclerotigenum*	2	ND	*Hordeum vulgare* leaf	Iran	
739.69 MH424637, (MH427938)	*Acremonium sclerotigenum*	2	mucoid	humus-rich soil	Netherlands	
286.70D MH424629, (MH427928)	*Acremonium sclerotigenum*	2	mucoid	air in penicillin factory	Pakistan	
109041 MH424619	*Acremonium potronii*	3	mucoid	soft contact lens from inflamed human eye	Ontario, Canada	slow growth 37 °C
287.70O MH424659, (MH427954)	*Acremonium strictum*	3	mucoid	soil	Egypt	moderate growth 37 °C (9 mm/14 d)
Novicki UW836 AY138844	*Acremonium strictum genogroup II*	3	mucoid	human blood	Washington State, USA	ND
114224 MH424668, (MH427943)	*Acremonium sclerotigenum*	3	mucoid	human nail	Ontario, Canada	
114225 MH424669, (MH427942)	*Acremonium sclerotigenum*	3	mucoid	human nail	Ontario, Canada	
114226 MH424666, (MH427941)	*Acremonium sclerotigenum*	3	mucoid	human nail	Ontario, Canada	
114227 MH424662	*Acremonium sclerotigenum*	3	mucoid	human nail	Ontario, Canada	slow growth 37 °C
114229 MH424667, (MH427951)	*Acremonium sclerotigenum*	3	mucoid	human nail	Ontario, Canada	
OMH F1648.97 MH424663	*Acremonium sclerotigenum*	3	mucoid	human nail	Ontario, Canada	
OMH F2629.97 MH424665	*Acremonium sclerotigenum*	3	mucoid	human nail	Ontario, Canada	
UTHSC 01-194 MH424649, (MH427912)	*Acremonium strictum*	3	mucoid	human blood	Washington state, USA	
UTHSC 07-3739 MH424657, (MH427918)	*Acremonium alternatum*	3	mucoid	human toenail	Minnesota, USA	
UTHSC 05-2270 MH424655	*Acremonium strictum*	3	mucoid	human blood	Utah, USA	
UTHSC 02-2890 MH424651, (MH427914)	*Acremonium strictum*	3	mucoid	human olecranon bursa	Wisconsin, USA	
UTHSC 02-2054 MH424650, (MH427913)	*Acremonium alternatum*	3	mucoid	human tracheal aspirate	Ohio, USA	
UTHSC 08-3294 MH424658, (MH427915)	*Acremonium strictum*	3	mucoid	human sputum	California, USA	
149.55 MH424623, (MH427933)	*Acremonium sclerotigenum*	3	mucoid	diseased *Dianthus caryophyllus*	Netherlands	
FMR 10587 MH424647, (MH427919)	*Acremonium sclerotigenum*	3	mucoid	human nail	Tarragona Prov., Spain	
286.70B MH424627, (MH427929)	*Acremonium sclerotigenum*	3	mucoid	ND	Germany	

^1^ ND = no data.

**Table 3:** Additional isolates included in actin sequencing only or as outgroups.

Species and GenBank Record (Actin Unless Noted)	Isolate # (CBS Accession Number Unless Noted)	Habitat	Origin
*Acremonium sclerotigenum* MH427935	159.61 ^1^	ND ^2^	USA
*A. sclerotigenum* MH427944	113719	ND	Iran
*A. sclerotigenum* MH427945	395.70B	human toenail	Florida, USA
*A. sclerotigenum* MH427946	114228 ^1^	human toenail	Ontario, Canada
*A. sclerotigenum* MH427948	101572	Endophyte in grapevine	California, USA
*A. sclerotigenum* MH427949	476.96	stony coastal soil	Madang Prov., Papua New Guinea
*A. sclerotigenum* MH427950	692.86	ND	Isère Dept., France
*A. sclerotigenum* MH427952	120606	grapevine	Madera Cty., California, USA
*A. sclerotigenum* MH427953	120819	grapevine	Madera Cty., California, USA
*Ovicillium attenuatum* KU382189 ITS	112092	*Theobroma*	Ecuador
*Ovicillium oosporum* KU382203 ITS	101236	Lepidoptera larva	Brazil
*Emericellopsis terricola* (T) U57676 ITS	120.40	soil	Netherlands
*Acremonium tubakii* (T) MH424671 ITS	790.69	seacoast	Wakayama Pref., Japan
*Emericellopsis maritima* NR*_*144919 ITS *Emericellopsis pallida* NR_145052 ITS *Emericellopsis alkalina* NR_145051 ITS *Stilbella fimetaria* KX446764 ITS	491.71 490.71 127350 AUMC 11023	seawater seawater ND salt lake	Crimea Crimea ND Behira Gov., Egypt
*Acremonium spinosum* (T) HE608629	136.33	human nail	Argentina
*Acremonium curvulum* (T) HE608630 MH424698 ITS	430.66	soil	Schleswig-Holstein, Germany

^1^ CBS 159.61 and 114228 cluster with members of ITS barcode group 3 in actin analysis. ^2^ ND = no data.
